# Development of predictive nomogram for clinical use of special-grade antimicrobial agents in patients with diabetes foot infections

**DOI:** 10.3389/fendo.2025.1578767

**Published:** 2025-08-12

**Authors:** Qian Wang, Hui Ma, Qiang Jiang, Lubo Guo

**Affiliations:** ^1^ Department of Pharmacy, Central Hospital Affiliated to Shandong First Medical University, Jinan Central Hospital, Jinan, Shandong, China; ^2^ Department of Endocrinology, Central Hospital Affiliated to Shandong First Medical University, Jinan Central Hospital, Jinan, Shandong, China

**Keywords:** diabetes foot infections, special-grade antimicrobial agents, Wagner grade, multivariate logical regression analysis, nomogram model

## Abstract

**Objective:**

To develop a predictive model to quantify the possibility of special-grade antimicrobial agents (SGAs) usage in patients with diabetes foot infections (DFIs), providing reference and guidance for clinical practice.

**Methods:**

This is a cross-sectional study of 328 type 2 diabetes patients with DFIs. General clinical characteristics and biochemical indicators were extracted from the Hospital Information System (HIS) of Jinan Central Hospital in Shandong Province, China. Logistic regression analysis was performed to select predictors, and the nomogram was established based on selected viables visually. Then, the receive operating characteristic (ROC) curve, the calibration curve and the decision curve analysis (DCA) were used to evaluate the performance of this prediction model.

**Results:**

5 predictors were selected by univariate analysis from 21 variables, including duration of hospitalization, Neutrophil, DBIL, ALB and Wagner grade. The multivariate logical regression analysis illustrated that these 5 factors were independent risk factors for SGAs usage in patients with DFIs. The nomogram model developed by these 5 risk predictors exhibited good prediction ability, as shown by the area under curve (AUC) of ROC curve was 0.884 in the training set and 0.825 in the validation set. Calibration curve showed a good calibration degree of the predictive nomogram model. Moreover, DCA curve showed that the nomogram exhibited greater clinical application values when the risk threshold was between 3% and 63%.

**Conclusion:**

Our novel nomogram model showed that duration of hospitalization, Neutrophil, DBIL, ALB and Wagner grade were the independent risk factors of SGAs usage in patients with DFIs. This prediction model behaved a great accurate value and provide reference of SGAs usage in clinic. Further validations are still needed to evaluate and improve the performance of this model.

## 1 Introduction

As a common epidemic metabolic disease in the 21st century, the prevalence of diabetes is exploding all over the world. According to statistics, more than 550 million people have diabetes worldwide (1). It is estimated that 783.2 million people will have diabetes in 2045 (2). Diabetic foot (DF) is one of the most serious and devastating complications in diabetic patients. Currently, approximately 20 million patients with diabetes suffer from foot ulcer annually (3), which has become one of the leading cause of disability. Moreover, about 50% of diabetic foot ulcers (DFUs) become infected (4), and it can lead to amputation and even death without appropriate care. Thus, the management of DFIs is extremely important for improving the outcomes of diabetes patients.

Multiple interventions are typically used to heal DFIs, such as management of infection. Antimicrobial agents treatment is one of main strategy to deal with all kinds of infections, including DFIs. However, the overuse and misuse of antimicrobial agents in the past decades contributes to the emergence of resistant microbials (5–7). To ensure the rational use and avoid the misuse of antimicrobial agents, China formally implemented a decree for the clinical use of antimicrobial agent in 2012. According to this decree, antimicrobial agents were classified as non-restricted, restricted and special-grade (8). It’s noteworthy that the SGAs, with strongest antimicrobial activity, has the strictest rules for prescription and use. However, it’s unclear what state of the patients should be administrated with the SGAs in clinic. And there is no predictive model to estimate the use of SGAs.

Considering these challenges, our study tended to develop a predictive model to quantify the possibility of SGAs usage in patients with DFIs. From this point of view, we hope to provide reference and guidance for the prescription and use of SGAs in DFIs patients clinically.

## 2 Methods

### 2.1 Study participants

This study was a cross-sectional study. 328 type 2 diabetes patients with DFIs (hospitalized in Jinan Central Hospital from January 2017 to December 2022) were included in this study. The inclusion criteria were as follows: (1) all participating patients were confirmed diagnosis of DFIs declared by WHO in 1990. (2) all patients were at least 18 years old. The exclusion criteria were as follows: (1) patients with other infections except diabetes foot, (2) patients with severe organic disease, such as malignant tumor, etc. (3) patients with serious lack of clinical data.

### 2.2 Data collection

We collected the clinical data of all patients from the HIS of Jinan Central Hospital, including general information, disease information and the blood biochemical indicator examination results. General information included sex, age, duration of hospitalization, course of diabetes, smoking history, drinking history, cardiovascular history (hypertension, cardiopathy, coronary heart disease, etc.). Blood biochemical indicators examination included γ-glutamyl transferase (GGT), alkaline phosphatase (ALP), creatinine (Crea), hemoglobin A1C (HbA1C), total bile acids (TBA), total bilirubin (TBIL), indirect bilirubin (IBIL), direct bilirubin (DBIL), albumin (ALB), urea (Urea). In addition, neutrophil counts (Neutrophil) and platelet (PLT) derived from peripheral blood samples were collected. The severity of DFUs was evaluated according to Wagner classification (9) (grade 0-5). The Wagner system was assessed based on the following grades: 0, pre- or post-ulcerative lesion; 1, partial or full-thickness ulcer; 2, probing to tendon or capsule; 3, deep with osteitis; 4, partial foot gangrene; 5, whole foot gangrene (9, 10).

### 2.3 Statistical analysis

To explore the influence factors of usage of SGAs, we divided the 328 patients into regular-grade antimicrobials agents (including non-restricted and restricted antimicrobial agents) (RGAs) group and SGAs group. The grade of all kinds of antimicrobial agents is referred to the Shandong Province Classification Management Catalogue for Clinical Application of Antibiotics (2021) in China (11). Then 328 patients were randomly divided into a training cohort and a validation cohort conformed to a ratio of 7:3 for random split validation. Data normality was analyzed by Kolmogorov-Sminov test. Continuous variables were expressed as median ± standard deviation (SD) and tested by t-test. The categorical variables were described as number (percentage) and analyzed by chi-squared test.

Univariate and multivariate logistic regression analysis were used to identify the influence factors for usage of SGAs in patients with DFIs. Odds ratios (ORs) were calculated with the 95% confidence intervals (95% CIs). P < 0.05 (two-sided) was considered as significant. According to the independent influence factors for usage of SGAs in patients with diabetic mellitus foot, we developed a nomogram prediction model. In addition, several validation methods were performed to evaluate the accuracy of the nomogram prediction model based on the data sets of training cohort and the validation cohort, respectively. AUC of ROC curve was employed to estimate the performance of the model. The calibration curve and the DCA was used to assess the calibration performance and clinical practicability of the prediction model, respectively. All statistical analysis was performed using IBM SPSS Statistics 26.0 and R software (version 3.6.1).

### 2.4 The list of SGAs and RGAs

The SGAs mainly include lenampicillin, sulbactam, cefepime, ceftazidime and avibactam, meropenem, imipenem and cilastatin, biapenem, ertapenem, aztreonam, flomoxef, tigecycline, sitafloxacin, vancomycin, norvancomycin, teicoplanin, ploymyxin B, polymyxin E, fusidic acid, linezolid, daptomycin, amphotericin B, voriconazole (for injection), itraconazole (for injection), caspofungin and micafungin.

The RGAs mainly include penicillin, amoxicillin, ampicillin, piperacillin, mezlocillin, azlocillin, oxacillin, cloxacillin, flucloxacillin, amoxicillin and clavulanate, ampicillin and sulbactam, mezlocillin and sulbactam, piperacillin and tazobactam, ticarcillin and potassium, cephalosporins (generation 1-4) except cefepime, cefoperazone and sulbactam, cephamicins, latamoxef, faropenem (oral agent), sulphonamides, trimethoprim, macrolides, lincosamides, aminoglycosides, tetracyclines except tigecycline, chloramphenicol, quinolones except sitafloxacin, colistin, nitroimidazole derivatives, nitrofuran derivatives, fosfomycin, rifampicin, rifaximin, rifamycin, fluconazole, nystatin, voriconazole (oral agent), itraconazole (oral agent), flucytosine, posaconazole (oral agent), terbinafine.

## 3 Results

### 3.1 Participant characteristics of the study cohort

Of the 328 participants enrolled in this study, with a median age of 74.0 years (40.0-96.0), of whom 116 (35.4%) were women and 212 (64.6%) were men. Of these 328 patients, 53 (16.16%) were administrated with the SGA, and 275 (83.84%) were administrated with the RGA. The median course of diabetes was 16.0 years.

The basic characteristics of all patients were shown in **Table 1**. Among these patients, 124 had a history of smoking, 122 had a history of drinking and 295 had cardiovascular history. The median Neutrophil was 5.07 x 10^9^/L, and the median ALB was 37.4 g/L. There was 179 patients whose Wagner grade less than 3, and 149 not. The clinicopathologic characteristics were shown in **Table 1**.

**Table 1:** Participant characteristics of the study cohort.

Variables	All patients (n=328)
Antimicrobial grade	
RGA	275 (83.84%)
SGA	53 (16.16%)
Age (years)	74.0 [40.0, 96.0]
Sex	
Women	116 (35.4%)
Men	212 (64.6%)
Course of diabetes (years)	16.0 [0.00, 40.0]
Duration of hospitalization (days)	14.0 [1.00, 170]
Smoking history	
NO	204 (62.2%)
YES	124 (37.8%)
Drinking history	
NO	206 (62.8%)
YES	122 (37.2%)
Bedridden	
NO	192 (58.5%)
YES	136 (41.5%)
Cardiovascular history	
NO	33 (10.1%)
YES	295 (89.9%)
Neutrophil (x10^9^/L)	5.07 [1.53, 24.1]
PLT (x10^9^/L)	227 [73.0, 721]
GGT (IU/L)	20.0 [5.00, 433]
ALP (IU/L)	78.0 [25.0, 597]
Crea (μmol/L)	75.0 [0.00, 925]
HbA1C (%)	7.90 [4.40, 16.0]
TBA (μmol/L)	3.60 [0.400, 359]
TBIL (μmol/L)	8.90 [2.00, 65.6]
IBIL (μmol/L)	4.50 [0.300, 23.2]
DBIL (μmol/L)	3.95 [0.700, 51.0]
ALB (g/L)	37.4 [20.8, 52.3]
Urea (nmol/L)	5.90 [1.50, 37.5]
Wagner grade	
< 3	179 (54.6%)
≥ 3	149 (45.4%)

### 3.2 Logistic regression variable screening results

All patients were divided into RGA group and SGA group based on the usage of antimicrobials. The results of univariate analysis presented significant differences in duration of hospitalization, bedridden, Neutrophil, PLT, GGT, ALP, DBIL, ALB, Urea, and Wagner grade (**Table 2**). Given the bedridden, PLT, GGT, ALP and Urea weren’t highly significant between RGA group and SGA group (P > 0.01), we exclude them and put the other highly significant parameters into multivariate logistic regression analysis (**Table 3**). The results represented that among our patients with T2DM, duration of hospitalization, Neutrophil, DBIL, ALB and Wagner grade were independent risk factors for SGAs usage.

**Table 2:** Differences in characteristics between RGA group and SGA group.

Variables	RGA(n=275)	SGA(n=53)	*χ^2^/t*	*P*
Age (years)	73.9 (12.2)	73.8 (11.2)	0.045	0.999
Sex			1.041	0.307
Women	94 (34.2%)	22 (41.5%)		
Men	181 (65.8%)	31 (58.5%)		
Course of diabetes (years)	17.8 (8.88)	15.7 (10.7)	1.513	0.0775
Duration of hospitalization (days)	14.9 (9.16)	27.8 (24.6)	-3.781	<0.001
Smoking history			0.367	0.544
NO	173 (62.9%)	31 (58.5%)		
YES	102 (37.1%)	22 (41.5%)		
Drinking history			0.049	0.825
NO	172 (62.5%)	34 (64.2%)		
YES	103 (37.5%)	19 (35.8%)		
Bedridden			6.044	0.0145
NO	169 (61.5%)	23 (43.4%)		
YES	106 (38.5%)	30 (56.6%)		
Cardiovascular history			1.350	0.245
NO	30 (10.9%)	3 (5.7%)		
YES	245 (89.1%)	50 (94.3%)		
Neutrophil (x10^9^/L)	5.54 (2.95)	9.85 (5.27)	-5.790	<0.001
PLT (x10^9^/L)	239 (89.0)	274 (107)	-2.248	0.0247
GGT (IU/L)	32.2 (47.9)	37.7 (33.7)	-0.795	0.0154
ALP (IU/L)	85.2 (41.1)	105 (79.3)	-1.790	0.0142
Crea (μmol/L)	117 (135)	133 (160)	-0.775	0.947
HbA1C (%)	8.25 (1.95)	8.59 (2.03)	-1.137	0.297
TBA (μmol/L)	4.67 (3.59)	11.5 (49.2)	-1.006	0.226
TBIL (μmol/L)	9.51 (5.01)	12.4 (10.6)	-1.946	0.144
IBIL (μmol/L)	5.15 (3.25)	5.06 (3.84)	0.178	0.345
DBIL (μmol/L)	4.36 (2.19)	7.35 (8.47)	-2.548	0.0019
ALB (g/L)	38.0 (5.74)	32.9 (5.86)	5.981	<0.001
Urea (nmol/L)	7.57 (5.81)	9.36 (6.51)	-2.012	0.0195
Wagner grade			31.814	<0.001
< 3	168 (61.1%)	11 (20.8%)		
≥ 3	107 (38.9%)	42 (79.2%)		

**Table 3:** Multivariate logistic regression analysis of DF.

Variables	*β*	*SE*	*Waldχ^2^*	*OR*	*95% CI*	*p*
Duration of hospitalization (days)	0.053	0.015	12.698	1.055	[1.024,1.086]	<0.001
Neutrophil (x10^9^/L)	0.138	0.049	7.911	1.148	[1.043,1.264]	0.005
DBIL (μmol/L)	0.088	0.042	4.422	1.092	[1.006,1.186]	0.035
ALB (g/L)	-0.072	0.035	4.184	0.931	[0.869,0.997]	0.041
Wagner grade	1.264	0.426	8.820	3.539	[1.537,8.147]	0.003

### 3.3 Nomogram prediction model construction and validation

**Figure 1:**
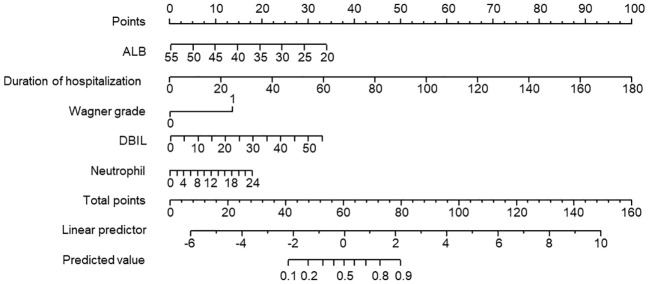
Development of the risk nomogram prediction model.

The 5 prediction variables obtained from the logistic regression analysis were incorporated to generate the nomogram prediction model, as shown in **Figure 1**. The individual variable was projected to obtain the corresponding score and the total score added by all individual scores of each factor was calculated. Then the risk probability could be predicted based on the total score by a vertical line across the total score point line.

**Figure 2:**
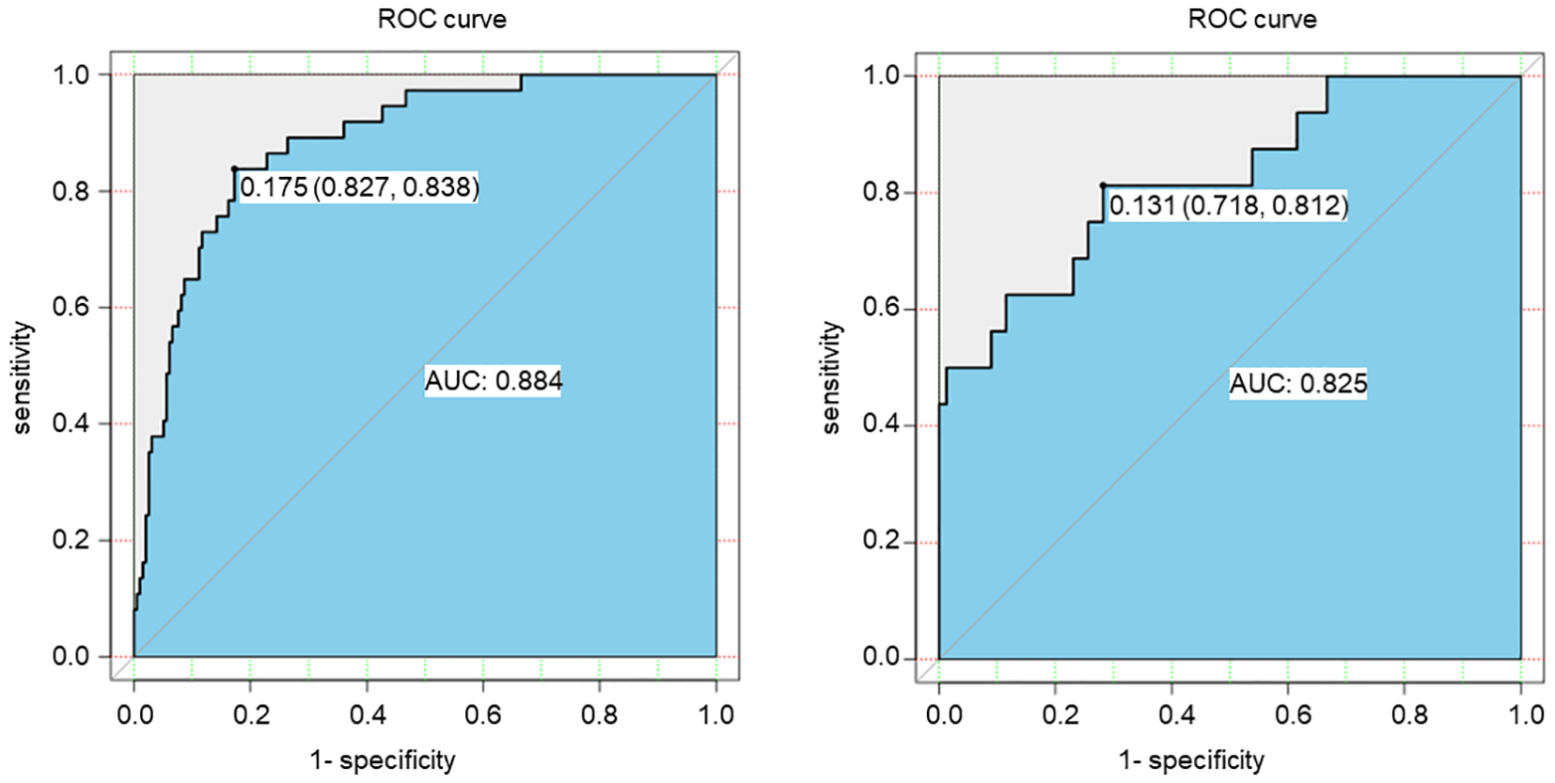
ROC curve of the risk prediction model for SGAs usage in patients with DF based on the training set (left) and the validation set (right).

The ROC curve was conducted to assess the discriminatory capacity of the nomogram model. The AUC of the prediction model was 0.884 in the training set and 0.825 in the validation set, indicating the good accuracy (**Figure 2**).

**Figure 3:**
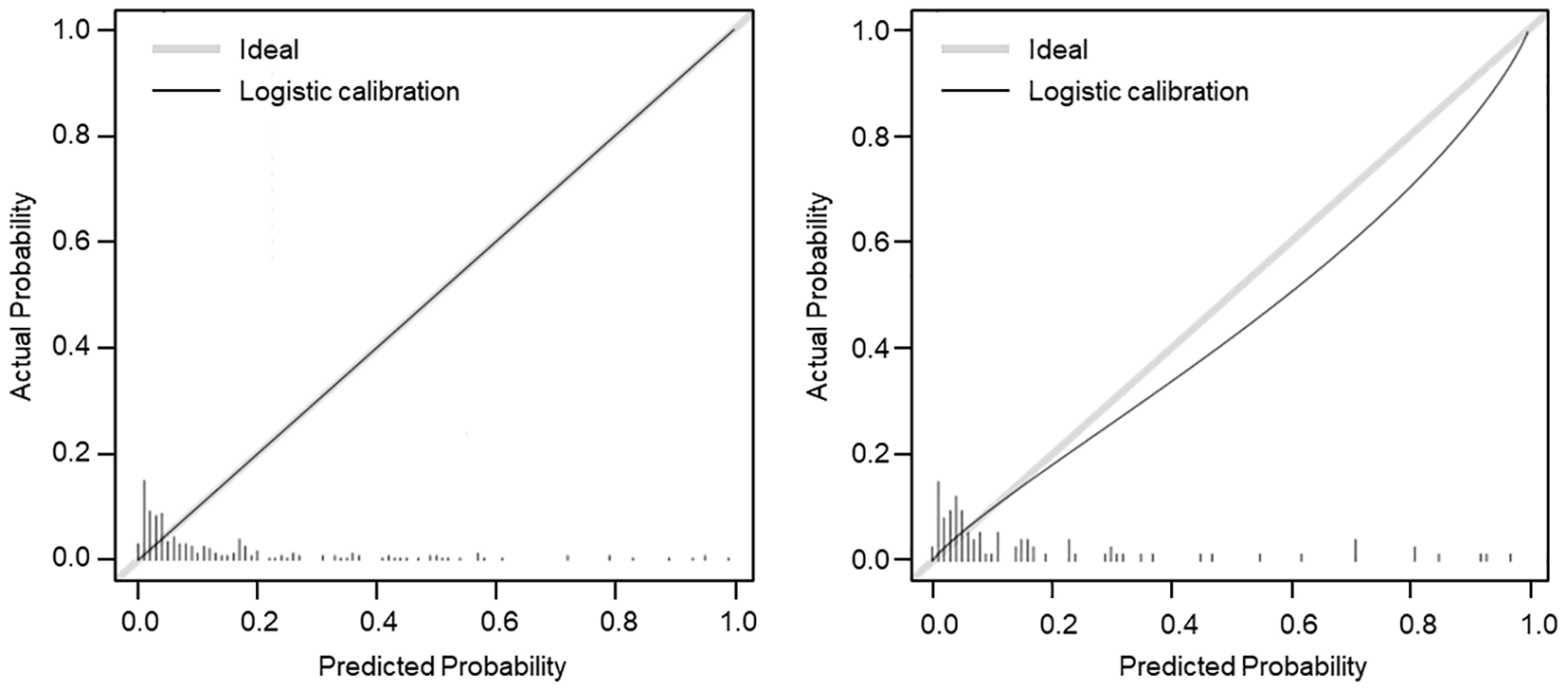
Calibration curve (β = 1000 repetitions, boot) of the SGAs usage risk nomogram. The x-axis represents the predicted probability of SGAs usage, and the y-axis represents the actual probability of SGAs usage. The gray dashed line represented the ideal curve, and the black line of the training set (left) and validation set (right) represented the predictive performance of SGAs usage nomogram. The closer the black line is to the gray dashed line, the better predictive accuracy of the nomogram model.

The calibration curve was conducted to calibration the SGAs usage nomogram model. The calibration curve of the nomogram showed good mostly agreement (**Figure 3**), indicating good calibration degree of the predictive model.

**Figure 4:**
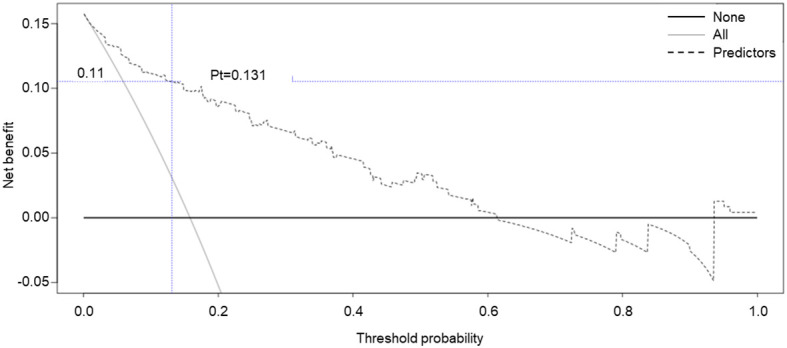
Decision curve analysis for the SGAs usage risk nomogram. The y-axis represents the net benefit.

Moreover, decision curve analysis (DCA) was further applied to validate the clinical benefits of the prediction model. The nomogram showed greater clinical application values when the risk threshold was between 3% and 63% (**Figure 4**), further demonstrating the good predictive and accuracy values of the model.

## 4 Discussion

As one of the most common complications of diabetes, DFUs is associated with impaired physical function and reduced quality of life (12, 13). If untreated, DFUs can progress to DFIs (14), which is significantly more likely to increased morbidity and cause death of patients with diabetes. DFIs are usually polymicrobial, and many microbials show resistance profiles to conventional antimicrobials resulted from the overuse and misuse of antimicrobial agents in the past decades. Given the abovementioned reasons, SGAs, such as Meropenem, are increasingly used for treatment of DFIs. But in clinic, physicians and pharmacists are unclear the condition to use the SGAs. Also, relevant research that can predict the possibility of SGAs usage in patients with DFI have rarely been conducted.

To do this, in this study, we analyzed the differences between patients with DFIs administrated with RGAs and SGAs. Through the risk factor analysis, duration of hospitalization, Neutrophil, DBIL, ALB and Wagner grade were the independent factors for SGAs usage in patients with DFIs. Based on that, we developed a predictive model for the usage of SGAs in patients with DFIs using these five available variables. This predictive model suggested that longer duration of hospitalization, higher neutrophil, higher DBIL, lower ALB and higher Wagner grade were the key individual factors determining the risk of SGAs usage in patients with DFIs.

Infection is a most common cause of antimicrobial agents usage. Once infected, numerous immune cells are rapidly recruited to the infected sites to recognize and eliminate microbial pathogens. Among these immune cells, neutrophil plays a decisive role. At the onset of infection, neutrophil quickly migrates to the site of infection and destroy microorganisms by phagocytosis, release anti-microbial contents and promotion the recruitment of other immune cell. Therefore, neutrophil is associated with many kinds of infections. As reported in the previous study, neutrophil was a independent risk factor for sepsis in patients with urinary tract infection (15). It is also reported that absolute neutrophil count was a predictor of multidrug-resistant bacteria infection in the patients with biliary tract infection (16). Moreover, Yan et al. also observed that percentage of neutrophils was the risk factor of multidrug-resistant organisms in patients with diabetic foot infection. Here, we found that neutrophil was a risk factor of SGAs usage in patients with DFIs. Antimicrobials agents were usually used for the treatment of infections and multidrug-resistance is a common cause for the usage of SGAs. Thus, our finding also confirmed a close relationship between neutrophils and infection in diabetes patient population.

Albumin is abundant in blood and serum albumin level is a prognostic marker for complications in bacteria and fungal infections (17). Albumin could offer protection microcirculation and tissues from inflammation processes induced by infections (17). It has reported that reduced albumin is a risk factor for bacteria infection of patients carrying non-healing wounds (18). Consistently, Our study showed albumin was a risk factor for SGAs usage in patients with DFIs, which usually accompanied by bacteria or fungal infections.

Total bilirubin (TBIL) is composed of IBIL and DBIL. Serum IBIL is converted to DBIL by hepatic enzyme UDP-glucurony transferase 1A1 in liver. All the TBIL, IBIL and DBIL are traditional liver function index. However, many research has suggested that they have different clinical implications. Several trials have suggested that DBIL is more important than TBIL and IBIL for metabolic syndrome and stroke (19, 20). Notably, a study by Wang et al. found that elevated serum DBIL, not TBIL or IBIL, was associated with the increased risk of T2D (21). Moreover, it has reported that DBIL is associated with insulin resistance risk (22). Here, our study showed that DBIL was a risk factor of SGAs usage in patients with DFIs. Based on the above research, we speculate that higher DBIL may lead to the SGAs usage for DFIs by affecting blood glucose and insulin resistance. Certainly, research including more samples is needed to confirm this association.

Wagner’s classification, used for assess for ulcer depth, osteomyelitis and gangrene, is the most widely accepted grading system for evaluating lesions of diabetes foot (9, 10). A retrospective study reported that Wagner system is a good predictor of lower extremity amputation and in patients with DF (9). A meta-analysis also showed that Wagner grade (≥3) is a risk factor for recurrence of DFU in patients with DF (23). Here, we found that Wagner grade is a risk factor for the SGAs usage in patients with DFIs, further confirming a close relationship between Wagner grade and severity of foot lesions of diabetes patients.

Our study also has several limitations. Firstly, the model was constructed through data collection from HIS, which may result in selection bias due to potential confounders and exclusion of patients with serious lack of clinical data. Secondly, further multi-center research and external validation should be performed to improve the generalizability and clinical applicability.

## 5 Conclusion

In conclusion, after evaluating multiple variables, we demonstrated that duration of hospitalization, Neutrophil, DBIL, ALB and Wagner grade were the independent risk factors of SGAs usage in patients with DFIs. Based on that, we established a novel nomogram predictive model of the SGAs in patients with DFIs. This predictive model has good performance, exhibiting great accurate value and discrimination. Furthermore, we will actively explore the cooperation with the endocrinologists, infectious disease specialists and other relevant experts, focusing on integrating our nomogram model into existing clinical judgment, so as to provide reference and guidance for the usage of SGAs in DFIs patients clinically.

## Data Availability

The original contributions presented in the study are included in the article/supplementary material. Further inquiries can be directed to the corresponding author.
